# Clinical Cases in Medical Wards of Mercy Hospital—Typhoid Fever, Carcinoma, with Extraordinary Symptoms

**Published:** 1869-11

**Authors:** N. S. Davis

**Affiliations:** Professor of Clinical Medicine


### CLINICAL CASES IN MEDICAL WARDS OF MERCY HOSPITAL—TYPHOID FEVER, CARCINOMA, WITH EXTRAORDINARY SYMPTOMS.

BY N. S. DAVIS, M.D., Professor of Clinical Medicine.

CASE I. *September 29th, 1869.*—The case before you, gentlemen, is that of a laboring man, aged about 28 years, naturally spare in flesh and of nervous temperament. He came to me about four weeks since, in my office, complaining of the usual *initial* symptoms of an attack of typhoid fever. I directed him some medicine, and proper hygienic management, and saw or heard nothing more from him until he was brought into this ward of the hospital, about one week since. At the time of admission his case presented all the symptoms of a grave form of typhoid fever, in the advanced stage of its progress. His skin was dingy; countenance dull; lips retracted and dry, leaving the upper teeth covered with sordes; mouth and tongue dry; mind somnolent and sometimes wandering; skin dry and rough, and above the natural temperature; muscular movements unsteady and awkward; abdomen tympanitic and full; bowels moving five or six times per day, the discharges being dark-brown and thin; respirations 20 per minute and short, with dry bronchial rhonchi over both sides of the chest, and some dulness on percussion over the lower and posterior parts. The pulse was soft, quick, and varying from 120 to 130 per minute. If we suppose that at the time he called at my office, he was in the forming stage of the fever, it will be seen, that when admitted into the hospital, he was at the end of the third week of the disease, and the symptoms such as to render the prognosis doubtful.

The soft, frequent pulse, the mental dulness, the muscular unsteadiness, the dark hue of the lips and skin all indicate that profound typhoid condition, when the qualities of the blood and the properties of the tissues are both impaired, causing all the resulting actions in the economy, such as capillary circulation, secretion, nutrition, inervation, etc., to be performed feebly. In the more malignant cases of typhus and typhoid fevers, these alterations in the qualities of the blood and the properties of the tissues are sufficient to suspend the organic changes, and, consequently, to prove the direct cause of death. In addition to the general pathological conditions, there are important local changes in the viscera of the chest and abdomen. The dry, bronchial rhonchi over the whole anterior part of the chest, with dulness on percussion over the lower and posterior part, and the short inspirations, show that the bronchial mucous membrane is in a state of congestion, and the parenchyma of the lower lobes so occupied with hypostatic or passive infiltration, as to materially diminish the capacity of the lungs for air.

The condition of the lungs, of course, lessens the oxygenation and decarbonization of the blood, and thus indirectly increases the general impairment of function throughout all the organs. The tympanitic abdomen, with the frequent, thin, redish-brown, and copious discharges from the bowels, indicate, in this stage of the disease, extensive softening, and, perhaps, ulceration of the aggregated glands of the ilium and mesentery.

These local pathological conditions in the chest and abdomen are frequently the most dangerous developments, during the progress of this variety of fever; sometimes determining a fatal result in cases presenting only moderate primary changes in the blood and properties of the tissues. In the patient before us, at the time of his admission, the symptoms, as we have already described, indicate much general depression, with serious lesions, both in the chest and abdomen.

Hence, the special indications for treatment were, to sustain the general properties and functions, by plenty of good air and judiciously selected nourishment, and to administer such medicines as would relieve the congested condition of the bronchial mucous membrane, on the one hand; and on the other such as would arrest the process of softening and disintegration in the glands of the ileum and colon. The first object was secured by the size of the ward, its free ventilation, and the limited number of patients in it, and the feeding of the patient, animal broths, well salted, alternately with thin, sweet milk and wheat flour porridge. These articles of nourishment, given in small quantities, and at short intervals, are capable of being taken up by the absorbants and lacteals of the stomach and duodenum, leaving the smallest amount of fecal residue to pass over the diseased surface of the ilium and colon. To accomplish the second purpose, we gave one fluid drachm of the following mixture, every four hours:—

| | | |
|---|---|---|
| ℞. | Hydrochlorate Ammonia, | ʒiij. |
| | Tart. Ant. et Pot., | 2 grs. |
| | Sulph. Morph., | 3 grs. |
| | Syrup Glycirrhiza, | ℥iv. |

Mix.

To secure the third object we give one fluid drachm of the following emulsion, every four hours, alternately, with the foregoing prescription:—

| | | |
|---|---|---|
| ℞. | Ol. Terebinth., | ʒiij. |
| | Tinct. Opii, | ʒiij. |
| | Pulv. G. Acaccia, } āā, | ʒiv. |
| | White Sugar, } | |
| | Rub together, and add Mint Water, | ℥iij. |

Mix.

After these remedies had been used three days, the dry, bronchial rhonchi diminished, and were partially replaced by moist mucous rattles; the skin became less hot and dry; but the pulse remained weak and frequent, and the mind more wandering.

The emulsion was continued every four hours, and 10 drops of chloroform added to each dose. The use of the solution of hydrochlorate of ammonia, etc., was diminished to one dose, morning, noon, and evening. The same nourishment was continued as before. Four days have elapsed since any alteration was made in his treatment.

If you now examine the patient carefully, you will find the skin but little above the natural temperature, and more soft; the countenance more pale; the lips thin, and still somewhat retracted, but the sordes mostly gone from the teeth; the middle of the tongue dry and red, but moist and white along the margins; the respirations shorter and more frequent than natural, with a moderate development of mucous rhonchus over the anterior part of the chest, and some dulness on percussion over the lower and posterior part. (Here the class were required, individually, to auscultate the patient.) The pulse is 110 per minute, small and soft. The abdomen is only slightly tympanitic, but the intestinal discharges continue thin and light-brown, and average from three to five discharges in the 24 hours. You will readily perceive that some of the symptoms to which your attention has been called indicate improvement, while others point to a more doubtful prognosis.

For instance, the nearer approach to a natural condition of the skin, the less appearance of sordes on the edges of the lips and teeth, the moist condition of the margins of the tongue, and the lessening of morbid sounds in the chest all indicate the commencement of convalescence. But the continued weakness and frequeney of the pulse, with the quality and number of the intestinal discharges, indicate the continuance of a serious amount of disease in the ilio-cæcal portion of the alimentary canal. It happens not very unfrequently in the severer cases of enteric or typhoid fever, that all the general symptoms of fever subside, and convalescence ensues, while these patches of aggregated glands in the ilium, which had become softened or ulcerated during the progress of the fever, are still not cicatrized or much improved in texture. Your attention is called to the fact as one of much practical importance. If it be overlooked, and as soon as the patient appears otherwise convalescent, all remedies designed to exert a soothing influence on this part of the mucous membrane are withdrawn, and a liberal diet allowed, it will sometimes happen that the intestinal evacuations will gradually increase in frequency; and after a week of partial convalescence, the abdomen will again become tympanitic, the mouth dry, the pulse frequent and feeble, with rapid loss of strength, until a fatal result is reached. In a smaller number of cases, the general appearances of convalescence continue, but the patient does not improve in strength.

The bowels do not become regular, sometimes moving three or four times in succession, and then quiet 24 or 36 hours. After a time, varying from one to three weeks, they are suddenly attacked with acute pain, in some part of the abdomen, followed rapidly by abdominal distension, tenderness, and prostration. The pulse becomes very rapid and feeble; the countenance hippocratic; the skin covered with cold perspiration; and death follows in from 24 to 48 hours. These are cases in which some one of the patches of Peyer's glands remained unhealed, after the convalescence of the general fever; and instead of subsequent cicatrization, it slowly extended, until the coats of the intestine were perforated, inducing, suddenly, peritonitis and death. Many years since, a marked instance of this kind occurred in the person of a medical student in this city.

After an apparently mild course of typhoid fever he convalesced, continued to be up a part of each day, for a week, and began to go to the table for his meals, with other boarders, when he was attacked suddenly with fatal peritonitis, from a perforating ulcer in the intestine. In a much larger number of cases, however, patients convalesce from typhoid fever, while numerous places in the mucous membrane are in a state of partial or complete ulceration. They regain a fair degree of flesh and strength, and often attempt to resume attention to their ordinary work. But the intestinal evacuations never become regular. In some, there will occur from one to three or four thin, fecal discharges per day, constituting what might be styled a slight, chronic diarrhœa. This state of the system will continue, in some cases, many months; and, finally, the patients begin to lose flesh and strength, and slowly reach a stage of fatal exhaustion.

In other cases, the uncicatrized patches appear to be limited to the colon. The patients recover a fair degree of flesh, and resume attention to business, but their intestinal evacuations remain very irregular, usually going from two to four days without any discharge, and then have six or eight in a single day. It would seem that the peristaltic motion of the small intestines was impaired, and the fecal contents were carried forward only slowly; but so soon as they begin to accumulate in the colon, and come in contact with the patches of diseased membrane, an exaggerated motion is started, which does not stop until the whole canal is emptied, when it returns to its dormant state as before. Patients have come to me often with this state of the bowels, and on carefully inquiring into their history, I have traced them directly back to an attack of typhoid fever, which had occurred, sometimes, four or five years previously. I am thus particular in calling your attention to this point, because it is one of direct practical importance.

Careful attention to the state of the bowels, during the convalescence from typhoid fever, will save many patients from troublesome sequelæ. The patient before us gives plain evidence of commencing convalescence, but his bowels remain actively loose, and his pulse quick and feeble.

We shall, therefore, continue to give him the emulsion of turpentine and laudanum, every four hours, and feed him on sweet milk and wheat-flour porridge, until the intestinal discharges become more natural.

Case II. Mrs. ——, aged 40 years, native of Ireland, was brought to the hospital several weeks since, from Kansas, where she had been living with her husband for some time past. I do not call your attention to this case for the purpose of discussing either the pathology or treatment of the disease under which she is laboring, but simply to point out some features that are of rare occurrence. You perceive that the right side of the face and neck are much swollen, and the right arm and hand still more swollen, distending the skin until it is shining and tense to the ends of the fingers. As I remove the covering you see the same swelling occupying the whole of the right anterior half of the chest, from below the breast to the top of the shoulder and neck, and from the middle of the sternum to the right laterally to the anterior edge of the scapula.

This swelling, embracing the right anterior and lateral half of the chest, right arm, shoulder, and corresponding side of the neck and face, is simply œdementous, pitting, as you see, on pressure, and accompanied by no discoloration, except along the right side of the trachea where it is purplish, as though the extravasation of serum or water had been accompanied by some of the red corpuscles. The œdematous infiltation in the neck and over the upper end of the sternum is so great as to render both respiration and deglutition somewhat difficult, and causing her to remain in an upright or inclined position nearly all the time. On examining further, you find the right breast little more than its natural size, the skin looking corrugated, and the nipple drawn in, while to the touch it is hard as a stick, and immovably fixed to the ribs, presenting a good sample of schirrhus, or hard cancer, involving the whole breast, mammary gland, areolar tissue, and all. The left breast is larger, but moveable on the ribs, with hard lumps or nodules of schirrhus limited to the mammary gland. There are no enlargements of the lymphatic glands in either axilla, or along the clavicles externally. The patient is of a sanguino-lymphatic temperament, not emaciated, and the general functions of the system very well performed.

The chief peculiarity of this case, which distinguishes it from ordinary cases of cancer in the breast, is the extent and location of the œdema.

Schirrhus of the breast is often accompanied by enlargement and induration of the Lymphatic glands in the axilla and along the border of the pectoral muscle. And these glands sometimes press upon the nerves and bloodvessels to such an extent as to make the arm both painful and œdematous.

But in this case there are no tumors in the axilla, and none to be felt externally along the clavicle. Neither is the œdema limited to the arm, as is usual in such cases: but it extends to all that part of the right side of the chest, shoulder, neck and face, from which the blood is returned through the right subclavian vein. This would indicate the formation of some cancerous growth behind the first rib or its junction with the sternum, in such position as to obstruct that vessel. The tumefaction caused by the œdema, however, is such as to prevent any satisfactory examination of that region, either by palpation or auscultation. That the œdema is the result of direct obstruction of bloodvessels, either by pressure or emboli, is evident from its strictly circumscribed character.

When dropsical effusions take place from spanæmia or impoverishment of the blood, they are general, and are always influenced by gravity, and hence appear most prominent in the feet and legs first. But here the lower extremities are entirely free. The apparent obstruction of the subclavian vessels, in this case, and the increasing œdema in the neck, will probably cause life to terminate early, by complete suspension of respiration and deglutition. As the hour has expired, I will not detain you for any comments on the subject of treatment; for, in this case, medicine and surgery are alike powerless.

